# Supplementary material for: Usp22 Overexpression Leads to Aberrant Signal Transduction of Cancer-Related Pathways but Is Not Sufficient to Drive Tumor Formation in Mice
Source: Cancers (Basel). 2021 Aug 25;13(17):4276. doi: 10.3390/cancers13174276 (PMC8428332; doi:10.3390/cancers13174276)

SUPPLEMENTARY INFORMATION

USP22 overexpression leads to aberrant signal transduction of cancer-related pathways but is not sufficient to drive tumor formation in mice.

Xianghong Kuang[1, 2], Michael J. McAndrew[1, 2,3], Lisa Maria Mustachio[1, 2], Ying-Jiun C. Chen[1, 2], Boyko S. Atanassov[4], Kevin Lin[1, 2], Yue Lu[1, 2], Jianjun Shen[1, 2], Andrew Salinger[1, 2], Timothy Macatee[1, 2], Sharon Y. R. Dent [1, 2,*], Evangelia Koutelou[1, 2,*]

SUPPLEMENTARY FIGURE LEGENDS

Figure S1. Verification of the targeted insertion of USP22 transgene in the ROSA26 locus. A. Map of the base vector pCAGGS-LSL-Luciferase (a gift from Dr. Ming-Jer Tsai) was used as the parental vector for the generation of the pCAGGS-LSL-Flag-Myc-USP22 targeting construct. **B.** Graph of the targeting construct integration in the ROSA26 locus for the interpretation of the Southern blot bands. Blue bars show the 5’ and 3’ probe used for the analysis. **C**. Southern blot analysis of the targeted locus using genomic DNA from the 3 selected ES clones (A10, B6, D1), with the 5’ and 3’ probes described in the methods. **D.** Southern blot analysis of the targeted locus using genomic DNA from adult mice (+/+, LSL/+, LSL/LSL), with the same 5’ and 3’ probes described in the methods. **E.** PCR genotyping products across homologous arms, that resulted in 1564 bp and 1342 bp products for wild-type and knock-in alleles, respectively, as described in the methods.

Figure S2. USP22 conditional overexpression in mammary gland epithelium induces over-branching. A. Female Rosa26KI-USP22-LSL mice were crossed with male MMTV-cre mice in which the MMTV promoter drives the expression of USP22 transgene in mammary epithelial cells. **B.** Nulliparous females at 16 weeks were used to collect mammary glands that were stained in carmine alum. Representative images of mammary gland trees from a wild type and a heterozygote OE/+ female mouse are shown**. C.** Quantification of branching was done by counting the total number of branch nodes found in about ¼ branching area from lymph node to the ending point in each genotype (n= 12) and normalized as numbers of nodes per 6X 10^6 μm^2 areas. P value was calculated with unpaired t test.

Figure S3. Endogenous *Usp22* is mostly expressed in luminal epithelial cells and may play a role in mammary gland hormone response. **A.** scRNA-seq of MECs derived from

nulliparous, midgestation, lactation, and post-involution mammary glands identified 15 clusters of MECs as described in Bach et al., 2017. **B.** Relative expression of *Usp22* within each cluster type is shown colorimetrically with relative expression shown in the bar to the right. **C, D**. Dendrogram of clusters based on the log-transformed mean expression values of the 15 clusters. The red blocks show the cluster subtype in which *Usp22* is expressed based on the relative abundance of transcripts shown in D. *Usp22* gene expression was detectable primarily in luminal cells and is more pronounced in hormone-sensing progenitors (clusters C1, C2), differentiated hormone-sensing cells (clusters C4, C5, C6), and alveolar cells (clusters C8, C10) that are only present in pregnant females. **E.** Isolated primary MECs and stromal fibroblasts from 3.5-month-old wild type, *USP22 OE/+* or *USP22 OE/OE* female mammary glands were analyzed with immunostaining for the epithelial marker Cytokeratin 8 (CK8) and for the fibroblast marker Fibroblast-specific protein 1 (Fsp-1). Scale bar 100um. **F.** qRT-PCR analysis of total RNA isolated from primary MECs and stromal fibroblasts from 3.5-month-old wild type, *USP22 OE/+* or *USP22 OE/OE* female mammary glands to validate the expression of epithelial (Cdh1, CK8) and fibroblast (Fsp-1) specific markers.

Figure S4. Validation of differential expression of genes implicated in signaling cascades upon USP22 overexpression in mouse mammary epithelial cells. A, B, C, D. qRT-PCR analysis of total RNA isolated from primary MECs from 3.5-month-old wild type, USP22 OE/+ or USP22 OE/OE female mammary glands and from control and USP22 expressing nMuMG mouse epithelial cells to detect differential expression of genes involved in mitotic spindle regulation (A), WNT-beta catenin pathway (B), Estrogen early response (C), and PI3K-AKT pathway (D). **E.** Representative immunoblots using total protein extracts from control and USP22 expressing nMuMG mouse epithelial cells, showing increased levels of phospho-ERK, but not of phospho-AKT. **F.** Representative immunoblots using whole cell extracts from control and USP22 expressing nMuMG mouse epithelial cells, showing similar levels of H2B ubiquitination.

Figure S5. USP22 ubiquitous overexpression does not affect the overall survival of male or female mice. A, B. Kaplan – Meier curves of survival of males (A) and virgin females (B) of wild type, USP22 OE/+ or USP22 OE/OE show no significant differences between the different cohorts observed.

SUPPLEMENTARY MATERIALS AND METHODS

Southern blot hybridization

Genomic DNA samples (6 µg) from ES cells or mice tails were digested overnight in a 37 °C water bath with 30 units of restriction enzyme EcoR V HF (New England Biolabs) in 35 µl total volume of 1X CutSmart Buffer. 10 units of EcoR V HF enzyme were added to each sample for an additional 2-3 hours on the second day. Samples were then separated overnight at 26 V on 0.8% agarose gels in Tris-acetate-EDTA (TAE) buffer. The gels were denatured for 1 hour in 1.5 M NaCl/0.5 M NaOH, neutralized for 40 minutes in 0.1 M Tris-HCl (pH 7.5)/0.5 M NaCl, washed with 20× SSC, then transferred for 24 hours with 20× SSC on positively charged membranes (Roche). The membranes were UV-crosslinked, rinsed with water and air-dried. 5' and 3' probe template vectors were prepared with PCR reaction using mouse genomic DNA as a template, inserted into the pCR 2.1 TA cloning vector. Probes were then labeled with DIG-dUTP by PCR using the PCR DIG Probe Synthesis Kit (Roche). The membranes were prehybridized and hybridized with heat-denatured 5′ probe or 3′ probe overnight at temperatures calculated according to the manufacturer's instructions from the DIG High Prime DNA Labeling and Detection Starter Kit II (Roche). The washing buffer (0.2× SSC, 0.5% SDS) was pre-warmed to 68°C, and then the membranes were washed twice for 15 minutes each at 68°C while shaken. Next, the probe-bound DNA fragments were detected using the DIG High Prime DNA Labeling and Detection Starter Kit II and exposed with X-ray films to the probed membrane for 40 minutes to obtain the desired signal, following the manufacturer's instructions.

Immunofluorescence staining of primary mammary gland epithelial cells and phospho-H3 immunohistochemistry staining of mammary glands

Mammary gland primary epithelial cells were isolated as described above and seeded in chamber slides (Nunc) with MEBM medium. The cells were fixed with 4% paraformaldehyde for 15 minutes, permeabilized with 0.5% Triton X-100 for 15 minutes and blocked with 10% donkey serum for 1 hour at room temperature. After blocking, cells were incubated overnight at 4°C with primary antibodies. Alexa Fluor (AF) 568-(red, Molecular Probes, Eugene, OR) or AF488-conjugated secondary antibody (green, Molecular Probes) were used for one hour after washing with PBS. Following three washes with PBS slides were mounted with the VECTASHIELD mounting medium with DAPI (Vector Laboratories, Burlingame, CA). The fluorescence images were taken using the Leica DMI 6000B fluorescence microscope. For immunohistochemistry staining, whole mammary glands were fixed in 10% neutral buffered formalin for 24-48 h then transferred to 70% ethanol, dehydrated in an ethanol series to 100% ethanol, cleared in xylene substitute, and then infiltrated with paraffin. Samples were then embedded in paraffin, sectioned at 5 µm, and put onto slides for immunoperoxidase staining with an anti-phospho-H3 antibody (from Millipore).

qRT-PCR primers

Gene name	Forward Primer	Reverse Primer
Setd5	GCAGGACAACATATCAGGTGG	GGTGATGCTTGTAGGTGTGTG
Pdk4	CCTTCACCACATGCTCTTCG	CGGTTTTCTTGATGCTCGAC
Bmpr1b	CCCTCGGCCCAAGATCCTA	CAACAGGCATTCCAGAGTCATC
Esrrb	GGACTCGCCGCCTATGTTC	CGTTAAGCATGTACTCGCATTTG
Kcne3	ATGGAGACTTCCAACGGGACT	GCCCGACGATCCTCAGTTTG
Axin2	TGACTCTCCTTCCAGATCCCA	TGCCCACACTAGGCTGACA
Mapk4	TGGGCAGTGGACGATCTTC	CTTCCCGATGGTCCTTAGCAG
Map3k11	ATGGAGCCCTTGAAGAACCTC	CTGTTGCCTTCGGAGACCC
Elf3-2	GCTGCCACCTGTGAGATCAG	GTGCCAAAGGTAGTCGGAGG
Jag2	CTGTGCAGCGTGTTCAGTG	GTGTCCACCATACGCAGATAAC
Plcb1	GCCCCTGGAGATTCTGGAGT	GGGAGACTTGAGGTTCACCTTT
Slc19a2	GTGGCATCGTTACTGATACCC	TTCCACCGGAGGCTCATCTAA
Pik3r3	TACAATACGGTGTGGAGTATGGA	GAGTCATTGGCTTAGGTGGCT
CK8	ATCGAGATCACCACCTACCG	TGAAGCCAGGGCTAGTGAGT
FSP-1	GGAGCTGCCTAGCTTCCTG	GCTGTCCAAGTTGCTCATCA
Cdh1	TACACTGCCCAGGAGCCAGA	TGGCACCAGTGTCCGGATTA

Kuang et al., Figure S1

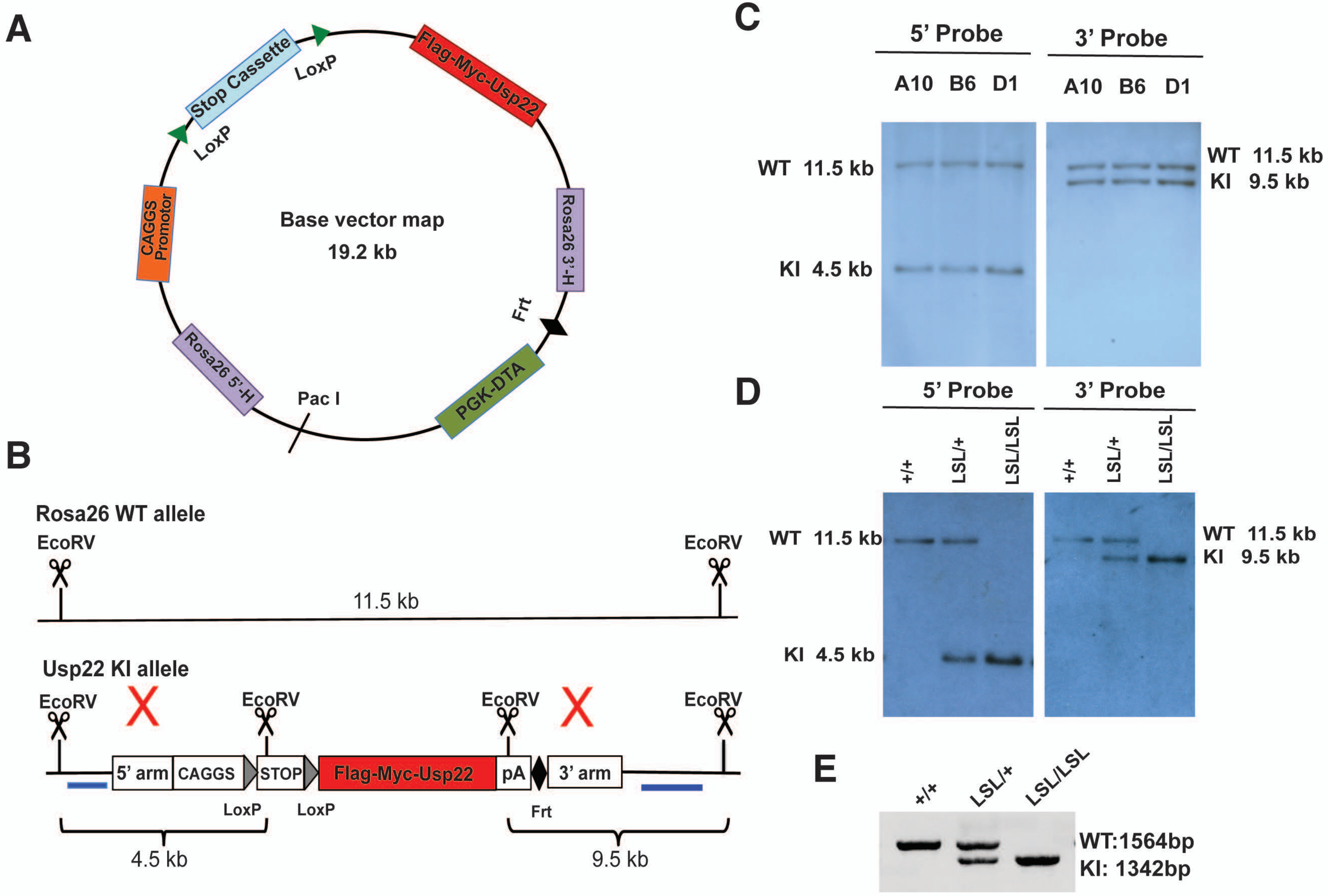

Kuang et al., Figure S2

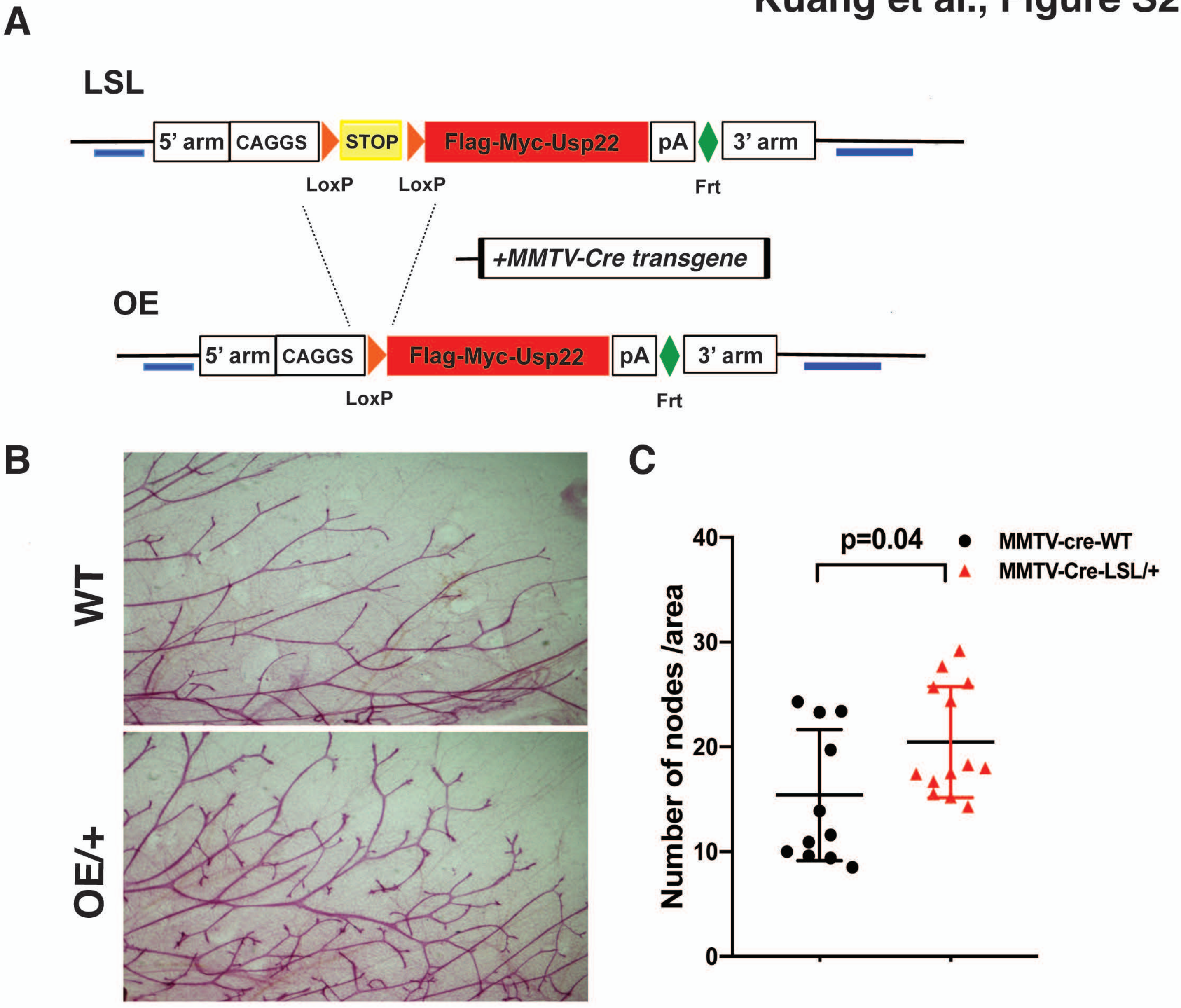

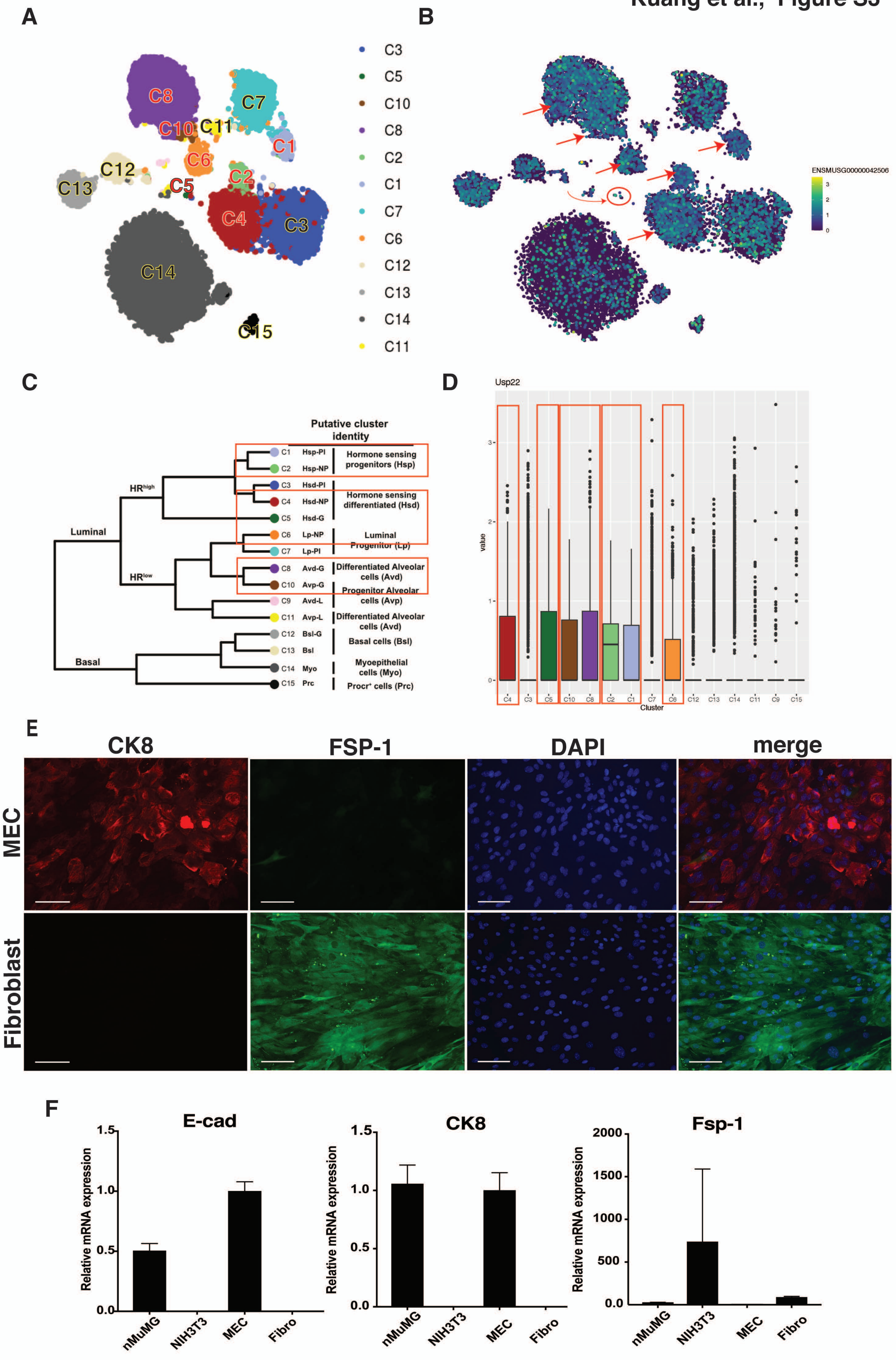
A
B
C
D
E
F
C3
C5
C10
C8
C2
C1
C7
C6
C12
C13
C14
C11
ENSMUSG00000042506
Putative cluster identity
Luminal
Basal
Hormone sensing progenitors (Hsp)
Hormone sensing differentiated (Hsd)
Luminal Progenitor (Lp)
Differentiated Alveolar cells (Avd)
Progenitor Alveolar cells (Avp)
Basal cells (Bsl)
Myoepithelial cells (Myo)
Procr+ cells (Prc)
Usp22
value
Cluster
CK8
FSP-1
DAPI
merge
MEC
Fibroblast
E-cad
Fsp-1
Relative mRNA expression
nMuMG
NIH3T3
Fibro

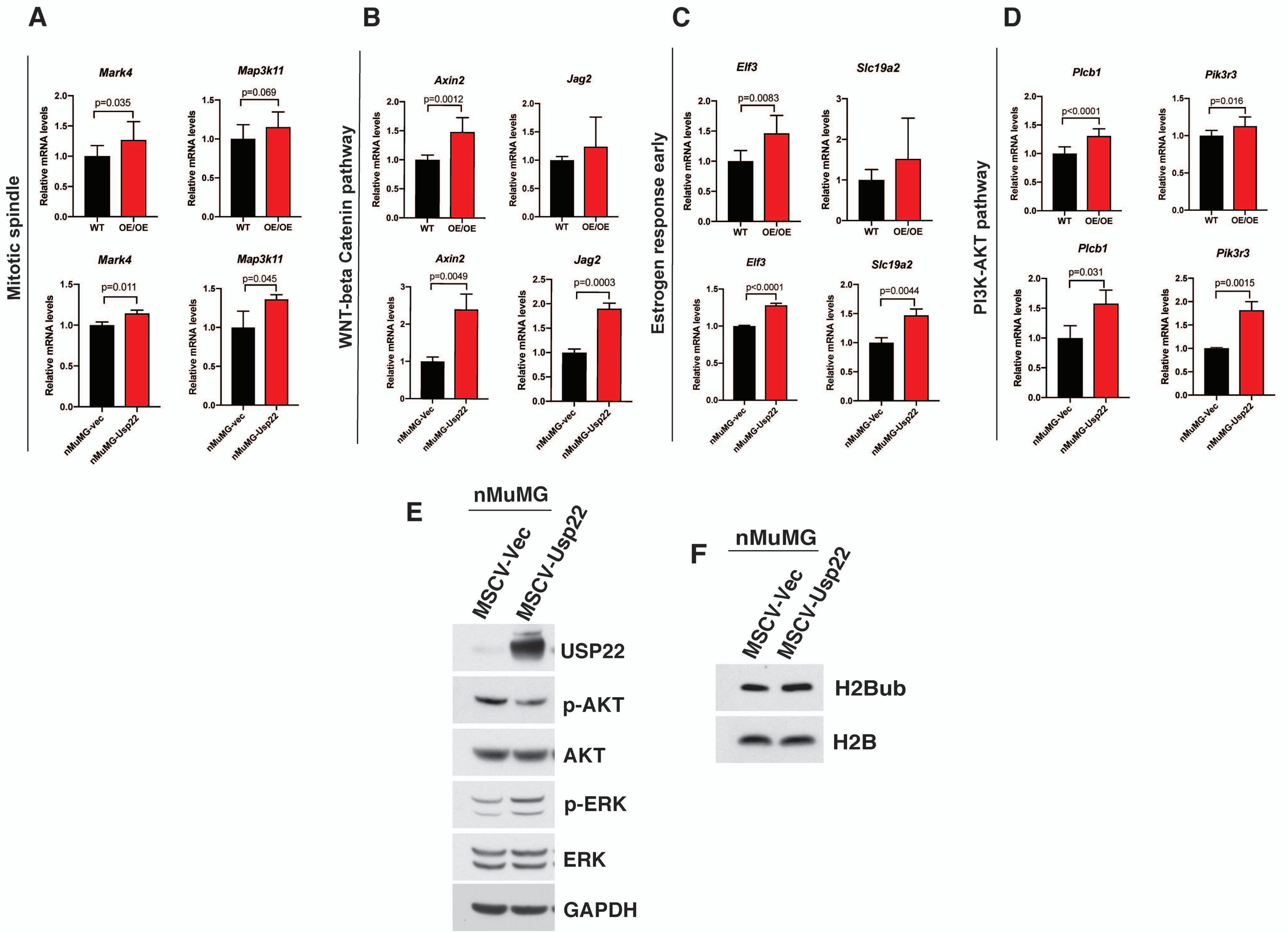
A
Mitotic spindle
Mark4
Map3k11
Relative mRNA levels
p=0.035
p=0.069
p=0.011
p=0.045
WT
OE/OE
nMuMG-vec
nMuMG-Usp22
B
WNT-beta Catenin pathway
Axin2
Jag2
p=0.0012
p=0.0049
p=0.0003
nMuMG-Vec
C
Estrogen response early
Elf3
Slc19a2
p=0.0083
p<0.0001
p=0.0044
D
PI3K-AKT pathway
Plcb1
Pik3r3
p<0.0001
p=0.016
p=0.031
p=0.0015
E
nMuMG
MSCV-Vec
MSCV-Usp22
USP22
p-AKT
AKT
p-ERK
ERK
GAPDH
F
nMuMG
MSCV-Vec
MSCV-Usp22
H2Bub
H2B

Kuang et al., Figure S5

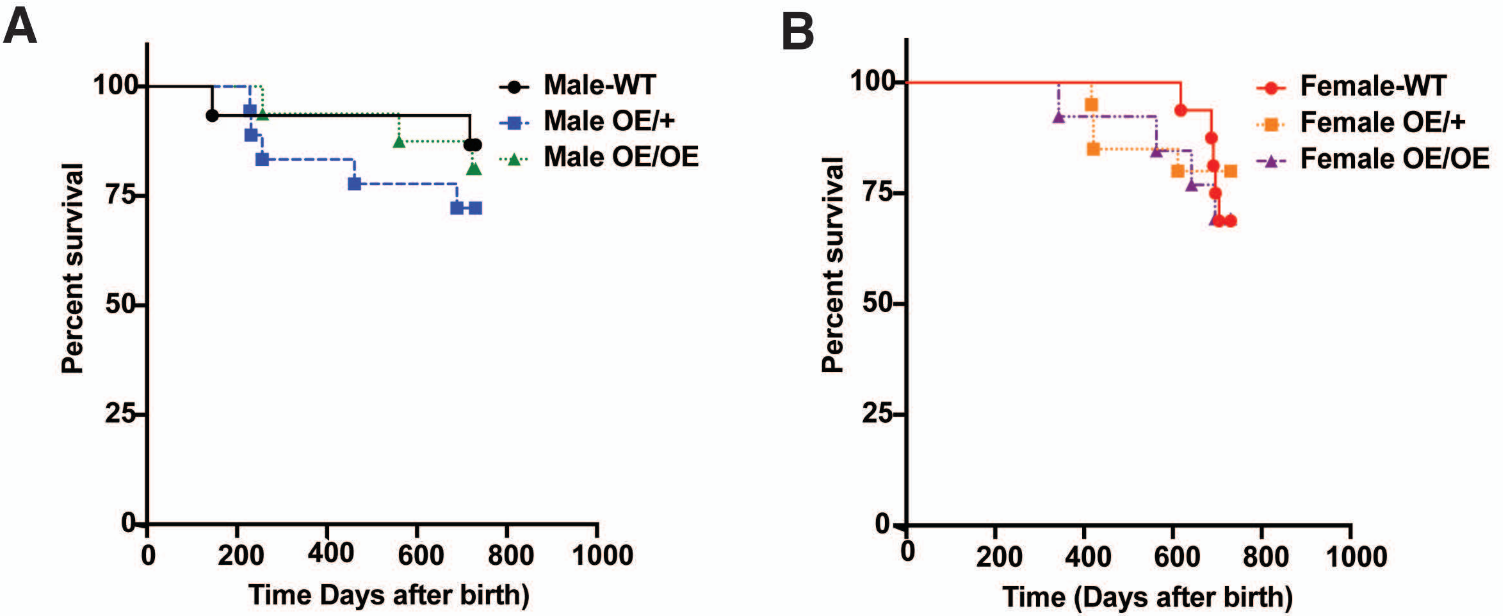

Supplement: Supplementary file 1 [file cancers-13-04276-s001.zip › cancers-1302237-supplementary.pdf]
